# Lipopolysaccharide (LPS) Aggravates High Glucose- and Hypoxia/Reoxygenation-Induced Injury through Activating ROS-Dependent NLRP3 Inflammasome-Mediated Pyroptosis in H9C2 Cardiomyocytes

**DOI:** 10.1155/2019/8151836

**Published:** 2019-02-17

**Authors:** Zhen Qiu, Yuhong He, Hao Ming, Shaoqing Lei, Yan Leng, Zhong-yuan Xia

**Affiliations:** ^1^Department of Anesthesiology, Renmin Hospital of Wuhan University, Wuhan, Hubei 430060, China; ^2^Office of Infection Control, Renmin Hospital of Wuhan University, Wuhan, Hubei 430060, China

## Abstract

Diabetes aggravates myocardial ischemia-reperfusion (I/R) injury because of the combination effects of changes in glucose and lipid energy metabolism, oxidative stress, and systemic inflammatory response. Studies have indicated that myocardial I/R may coincide and interact with sepsis and inflammation. However, the role of LPS in hypoxia/reoxygenation (H/R) injury in cardiomyocytes under high glucose conditions is still unclear. Our objective was to examine whether lipopolysaccharide (LPS) could aggravate high glucose- (HG-) and hypoxia/reoxygenation- (H/R-) induced injury by upregulating ROS production to activate NLRP3 inflammasome-mediated pyroptosis in H9C2 cardiomyocytes. H9C2 cardiomyocytes were exposed to HG (30 mM) condition with or without LPS, along with caspase-1 inhibitor (Ac-YVAD-CMK), inflammasome inhibitor (BAY11-7082), ROS scavenger N-acetylcysteine (NAC), or not for 24 h, then subjected to 4 h of hypoxia followed by 2 h of reoxygenation (H/R). The cell viability, lactate dehydrogenase (LDH) release, caspase-1 activity, and intracellular ROS production were detected by using assay kits. The incidence of pyroptosis was detected by calcein-AM/propidium iodide (PI) double staining kit. The concentrations of IL-1*β* and IL-18 in the supernatants were assessed by ELISA. The mRNA levels of NLRP3, ASC, and caspase-1 were detected by qRT-PCR. The protein levels of NF-*κ*B p65, NLRP3, ASC, cleaved caspase-1 (p10), IL-1*β*, and IL-18 were detected by western blot. The results indicated that pretreatment LPS with 1 *μ*g/ml not 0.1 *μ*g/ml could efficiently aggravate HG and H/R injury by activating NLRP3 inflammasome to mediate pyroptosis in H9C2 cells, as evidenced by increased LDH release and decreased cell viability in the cells, and increased expression of NLRP3, ASC, cleaved caspase-1 (p10), IL-1*β*, and IL-18. Meanwhile, Ac-YVAD-CMK, BAY11-7082, or NAC attenuated HG- and H/R-induced H9C2 cell injury with LPS stimulated by reversing the activation of NLRP3 inflammasome-mediated pyroptosis. In conclusion, LPS could increase the sensitivity of H9C2 cells to HG and H/R and aggravated HG- and H/R-induced H9C2 cell injury by promoting ROS production to induce NLRP3 inflammasome-mediated pyroptosis.

## 1. Introduction

Cardiovascular diseases, such as myocardial infarction, are considered to be one of the most important risk factors for death in modern social diseases [1]. Diabetes is an independent risk factor for myocardial ischemia-reperfusion (I/R) injury. Studies have shown that the incidence of myocardial I/R in diabetic patients is significantly higher than nondiabetic patients, and the diabetic patients had a larger infarct size and a higher new congestive heart failure rate than nondiabetic patients [2].

Sepsis is considered to be one most common cause of death in intensive care units, typically characterized by initial cytokine storm-induced inflammatory response followed by significant immunosuppression [3, 4]. Studies have shown that ventricular myocardial insufficiency during sepsis is accompanied by diastolic dysfunction [5]. Potential causes of septic cardiomyopathy include pathogen-associated molecular patterns (PAMP), such as lipopolysaccharide (LPS), lipoteichoic acid, cytokines, and nitric oxide [6, 7]. These mediators bind to pattern-recognition receptors (PRRs), and the interaction between PRRs and PAMPs can activate intracellular signal-transduction pathways that lead to nuclear translocation of nuclear factor-*κ*B (NF-*κ*B) and increase transcription of inflammatory mediators [8]. It is clear that the activation of innate immune system and resultant inflammatory response are important components of myocardial damage and I/R injury [9, 10]. Therefore, under the condition of sepsis, PAMP and DAMP stimulating factors are the causes of aggravated myocardial injury during myocardial I/R.

Pyroptosis is a recently identified type of programmed cell death. Caspase-1 activation is activated by inflammasomes (including NLRP3, NLRC4, Nlrp1b, and AIM2), which cleaves pro-caspase-1 into activated caspase-1, promoting the IL-1*β* and IL-18 precursors to form mature IL-1*β* and IL-18 then mediating pyroptosis, which plays an important role in the development and maintenance of inflammatory responses [11, 12]. The NLRP3 inflammasome is a nod-like receptor and could recognize diverse stimuli including PAMPs and DAMPs to activate pro-caspase-1 cleaves into form active caspase-1, then leads to maturation and secretion of IL-1*β* and IL-18 [13]. Studies have indicated that exogenous stimuli such as LPS and endogenous injury signals such as uric acid and ATP may induce common pathways (such as reactive oxygen species (ROS) production) to activate NLRP3 inflammasome and then trigger caspase-1-dependent pyroptosis [14, 15]. Studies have reported that LPS-mediated priming signal-induced NLRP3 mRNA expression is reduced by NF-*κ*B inhibitor or ROS inhibitor, indicating that NF-*κ*B and ROS play important roles in the regulation of NLRP3 gene expression [16, 17].

ROS is known to play a prominent role in the pathogenesis of various cardiac disorders, such as myocardial infarction (MI) and heart failure [18]. In diabetic state, the hyperglycemia, metabolic disorders, increased oxidative stress, and mitochondrial dysfunction could increase the production of ROS to aggravate myocardial damage [19, 20]. It has been reported that ROS is produced at an accelerated level in a few minutes after myocardial I/R, and diabetic patients have increased ROS production to exacerbate myocardial I/R injury [21]. As an intracellular target of ROS, NF-*κ*B plays a pivotal role in I/R injury, and inhibition of NF-*κ*B can protect myocardium from I/R injury [22]. Therefore, the production of ROS is an important target of myocardial I/R injury in diabetes.

Researches have clarified that ROS plays a role in inflammatory cytokine production in response to LPS [23, 24], which have also been shown to play an important role in activation of NLRP3 inflammasome [25, 26] and been considered as a potential mechanism to govern the increased sensitivity of diabetic heart to I/R injury [19]. What is more, our previous study has indicated that ROS activation-induced NLRP3 inflammasome triggering caspase-1-dependent pyroptosis plays an important role in MI/R injury in diabetic rats [27]. Whether LPS/ROS activated NLRP3 inflammasome is also involved in high glucose (HG) and hypoxia/reoxygenation (H/R) injury in cardiomyocytes is unclear. The relationship between LPS-induced cardiomyocytes injury and NLRP3 inflammasome-mediated caspase-1-dependent pyroptosis under HG and H/R stimulation is also unknown. In this study, we aim to investigate the role of LPS in regulating NLRP3 inflammasome-mediated caspase-1-dependent pyroptosis in H9C2 cells to aggravate HG and H/R injury and its underlying mechanism.

## 2. Materials and Methods

### 2.1. Cell Culture and Treatment Protocol

H9C2 rat cardiomyoblast cell line was purchased from the Cell Bank of the Chinese Academy of Sciences (Shanghai, China) and cultured in Dulbecco's modified Eagle medium (DMEM) (Gibco Laboratories, USA) supplemented with 10% fetal bovine serum (FBS) (Gibco Laboratories, USA) and 100 U/ml penicillin/100 mg/ml streptomycin in an atmosphere of 90% air and 10% CO_2_ at 37°C as described previously [27]. Medium was replaced every 2 days, and the cells were digested with 0.05% trypsin when the density of the cells reached 80–90%. H9C2 cells were seeded in six-well plates or 96-well plates and treated as used for the following experiments.

When the density of cells reached 50-60% in 6-well plates, the experimental group cells were incubated overnight with serum-free medium before treatment and then cultured at a final concentration of 30 mM glucose by 50% glucose injection for HG condition for 24 h in minimal essential medium with 1% FBS. After that, cells were subjected to a constant temperature three-gas incubator with a mixture of 95% N_2_, 5% CO_2_, and 1% O_2_ at 37°C for 4 h to establish hypoxia. Subsequently, plates were removed to a normoxic chamber for 2 h to establish reoxygenation.

### 2.2. Treatment of Cells

LPS (Sigma) was dissolved in sterile deionized water and used at a final concentration of 0.1 *μ*g/ml or 1 *μ*g/ml. The caspase-1 inhibitor Ac-YVAD-CMK (Sigma, USA) was dissolved in dimethyl sulfoxide (DMSO) and used at a concentration of 50 *μ*M. BAY11-7082 (Selleck, USA), as an inflammasome inhibitor, was dissolved in DMSO and used at a concentration of 5 *μ*M, and the ROS inhibitor N-acetylcysteine (NAC, Sigma, USA) was used at a concentration of 10 mM which dissolved in sterile deionized water.

### 2.3. Cell Viability Assay

Cell viability was determined by using a CCK-8 assay kit (Jiancheng, Nanjing, China) in 96-well plates according to the manufacturer's instructions. 10 *μ*l of CCK-8 reagent was added to the culture medium at each well and then continuously incubated for 3 h in darkness at 37°C. The absorbance was determined at 450 nm using a PerkinElmer microplate reader (PerkinElmer VICTOR 1420, USA).

### 2.4. LDH Activity Assay

LDH activity in the supernatants was measured for the evaluation of cell injury by using a commercially available LDH assay kit (Jiancheng, Nanjing, China) according to the manufacturer's instructions.

### 2.5. ELISA Assay

Culture supernatants were collected and the concentrations of IL-1*β* and IL-18 in the supernatants were assessed by enzyme-linked immunosorbent assay (ELISA) (Elabscience, China) according to manufacturer's instructions. The levels were normalized to cell protein concentrations.

### 2.6. Measurement of Caspase-1 Activity

The caspase-1 activity was assayed by using caspase-1 activity assay kit (Beyotime, China) according to the manufacturer's instructions. The absorbance was measured at a wavelength of 405 nm.

### 2.7. Calcein-AM/Propidium Iodide (PI) Staining

After different stimulation, the cells were collected by trypsinization into a cell culture medium, centrifuged at 1000 g for 5 minutes at room temperature to collect the cell pellet, and washed once with PBS. Then, the cells were washed twice with 1x assay buffer. After that, cells were mixed with 1x assay buffer and were stained with 2 *μ*M calcein-AM and 4.5 *μ*M PI per well at 37°C for 30 min. The images of the cells were acquired immediately and analyzed by using a fluorescence microscope (Olympus IX51, Japan). The percentage of positive cells was counted and the average fluorescence intensity was assessed with Image Pro advanced software.

### 2.8. Measurement of ROS Generation

Intracellular ROS level was assayed by the fluorescent probe dichloro-dihydro-fluorescein diacetate (DCFH-DA) (Sigma, USA). After the introduction of different stimulus, cells were incubated with 50 *μ*M DCFH-DA at 37°C for 30 min in the dark. Then, the cells were washed twice using cold PBS. The fluorescence images of intracellular ROS were acquired by using fluorescence microscopy (Olympus IX51, Japan). The average fluorescence intensity was analyzed by using an image analysis system (ImageJ, National Institutes of Health).

### 2.9. Immunofluorescence Staining

After different stimulation, H9C2 cells were fixed with 4% paraformaldehyde for 30 min, washed three times by PBS, and then treated with 0.2% Triton X-100 (Solarbio, China) at room temperature for 20 min. After being washed three times by PBS, cells were blocked in 5% BSA (Servicebio, China) for 30 min at room temperature. Subsequently, cells were incubated with primary antibodies against NLRP3 (1 : 50, Novus, USA) and caspase-1 (1 : 50, Santa Cruz, USA) overnight at 4°C. After that, samples were incubated with Cy3-conjugated anti-rabbit secondary antibody (1 : 300, Servicebio, China) at 37°C for 1 h. And then cells were stained with DAPI (Wuhan Antgene, China) for 5 min. Images were taken by fluorescence microscope (Olympus IX51, Japan).

### 2.10. Quantitative Real-Time PCR Analysis

Total RNA was extracted from cells using Trizol reagent (Invitrogen, USA) according to the manufacturer's instructions, and 1 *μ*g of total RNA was reversely transcribed into cDNA using a Reverse Transcription Kit (Takara, China). Primers were synthesized by Sangon Biotech (Shanghai, China). The mRNA levels of NLRP3, ASC, and caspase-1 were performed by quantitative RT-PCR (qRT-PCR) in 20 *μ*l reaction system containing specific primers and SYBR Green Master Mix (Takara, China) by CFX96 Real-Time PCR Detection System (Bio-Rad, USA). The levels of mRNA were normalized relative to GAPDH. The expression of genes was analyzed by using the 2^-△△CT^ method.

### 2.11. Western Blot Analysis

After treated with different stimulus, the cells were collected and lysed in ice-cold radio immunoprecipitation assay (RIPA) (Beyotime, China) buffer containing protease inhibitors phenylmethylsulfonyl fluoride (PMSF) (Beyotime, China) and protease inhibitor cocktail (Beyotime, China) and then centrifuged at 12000 rpm at 4°C for 15 min to obtain supernatants. Equal amount of protein lysates were loaded into a 5%-10-15% SDS-PAGE gel and transferred to polyvinylidene difluoride (PVDF) membrane. The membranes were blocked in 5% nonfat milk for 1 h at room temperature and then incubated overnight with primary antibodies against NF-*κ*B p65 (1 : 1000, CST, USA), NLRP3 (1 : 200, Novus, USA), ASC (1 : 200, Santa Cruz, USA), caspase-1 (1 : 200, Santa Cruz, USA), IL-1*β* (1 : 1000, Abcam, UK), IL-18 (1 : 1000, Abcam, UK), and GAPDH (1 : 1000, CST, USA). The membranes were subsequently incubated with fluorescent secondary antibody (1 : 15000, CST, USA) for 1 h at room temperature. Then, the membranes were washed again with TBST for 3 times, 5 minutes of each time. The protein bands were detected with an Odyssey color infrared laser scan-imaging instrument (LI-COR, USA). The images were analyzed using Odyssey Application Software 3.0.

### 2.12. Statistical Analysis

All data are represented as the mean ± SD. All statistical tests were performed by using GraphPad Prism version 6.0 (GraphPad Software, USA). One-way ANOVA or two-way ANOVA followed by Tukey's post hoc (a Bonferroni post hoc) test was performed to analyze the differences among experimental groups. *P* values < 0.05 were considered to be statistically significant.

## 3. Results

### 3.1. LPS Aggravated HG- and H/R-Induced Injury in H9C2 Cells

**Figure 1:**
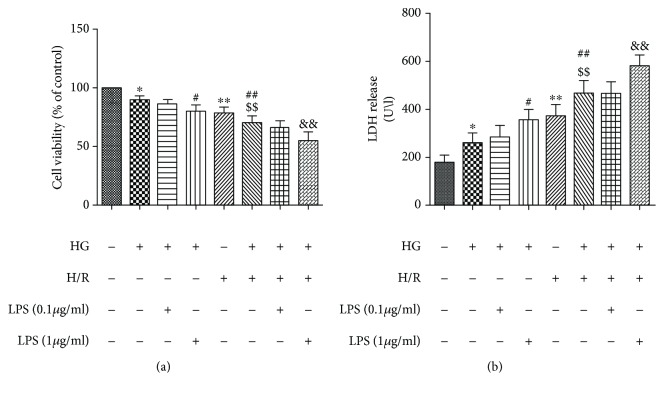
Dose-dependent effects of LPS on cellular viability and LDH release in H9C2 cells under HG and H/R condition. A “+” symbol indicates presence and a “−” symbol indicates absence of the relevant treatment condition. Cell viability was detected by CCK-8 assay (a). The LDH release was detected by LDH activity assays (b). Data are expressed as mean ± SD (*n* = 6). ^∗^*P* < 0.05 and ^∗∗^*P* < 0.01 versus control; ^#^*P* < 0.05 and ^##^*P* < 0.01 versus HG; ^$$^*P* < 0.01 versus H/R; ^&&^*P* < 0.01 versus HG+H/R.

To observe the effects of LPS on cellular activity by detecting the cell viability and LDH release, H9C2 cells were exposed to HG and H/R treatments along with 0.1 *μ*g/ml or 1 *μ*g/ml concentrations of LPS. As shown in Figures 1(a), the cell viability of HG+H/R group was significantly lower than those of control group and HG and H/R alone groups. The LDH release in HG+H/R group was also significantly increased than the control group and HG or H/R alone groups (Figures 1(b)). In addition, the concentration of 1 *μ*g/ml of LPS significantly decreased the H9C2 cell viability with higher LDH release under HG+H/R condition compared with the HG+H/R group and the low concentration 0.1 *μ*g/ml of LPS treated group (Figure 1), indicating that higher concentration (1 *μ*g/ml) of LPS could aggravate the HG+H/R-induced injury in H9C2 cells by increasing cytotoxicity. So, we used 1 *μ*g/ml LPS as an intervention condition in our subsequent experiments.

### 3.2. Dependence of Caspase-1 Activation-Mediated Pyroptosis in LPS Aggravated HG+H/R-Induced H9C2 Cell Injury

**Figure 2:**
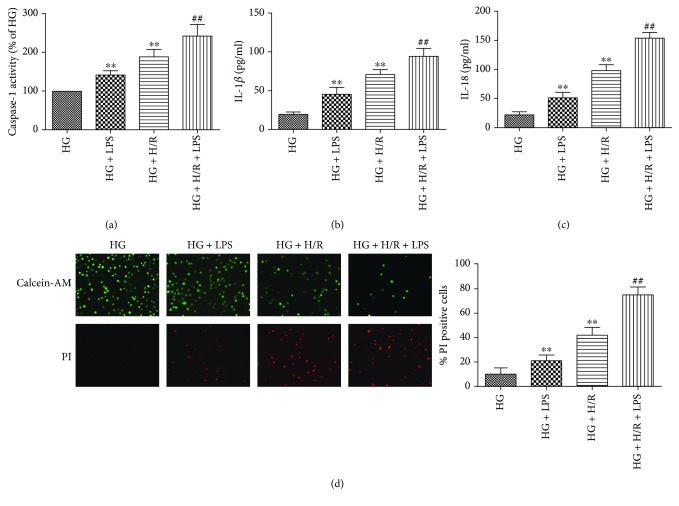
Effects of LPS on caspase-1 activity and pyroptotic cell death in H9C2 cells under HG and H/R stimulation. Caspase-1 activity was detected by caspase-1 activity assay kit (a). The levels of IL-1*β* (b) and IL-18 (c) in the supernatants were analyzed by ELISA. The pyroptotic cell death was detected by calcein-AM/PI staining assays (d). Data are expressed as mean ± SD (*n* = 6). ^∗∗^*P* < 0.01 versus HG; ^##^*P* < 0.01 versus HG + H/R.

LPS, the major exogenous stimuli, has been reported to induce ROS production and activation of caspase-1 and pyroptosis [24, 28]. To examine whether 1 *μ*g/ml LPS aggravated HG+H/R-induced H9C2 cell injury was involved in dependence of caspase-1 activation-mediated pyroptotic cell death, we tested the activity of caspase-1 of cells, IL-1*β* and IL-18 levels in the cell supernatants, and the positive cells of pyroptosis by calcein-AM/PI staining. Our results showed that the activity of caspase-1 was significantly increased in HG+LPS groups and HG+H/R groups compared with HG group and was further significantly increased in LPS+HG+H/R groups than HG+H/R groups (Figure 2(a)). As shown in Figures 2(b) and 2(c), the levels of IL-1*β* and IL-18 in HG+LPS groups and HG+H/R groups were increased compared with HG groups, and were further significantly increased in LPS+HG+H/R groups than HG+H/R groups. As shown in Figure 2(d), HG and H/R significantly induced pyroptotic cell death and further increased by LPS stimulation with increased pyroptotic positive cells and significantly increased in LPS+HG+H/R groups than HG+H/R groups. These results indicated that LPS induced the activation of caspase-1 and pyroptosis to aggravate HG- and H/R-induced H9C2 cell injury.

### 3.3. LPS Aggravated HG+H/R-Induced H9C2 Cell Injury by Increasing the Production of ROS and Expression of NLRP3 and Caspase-1

**Figure 3:**
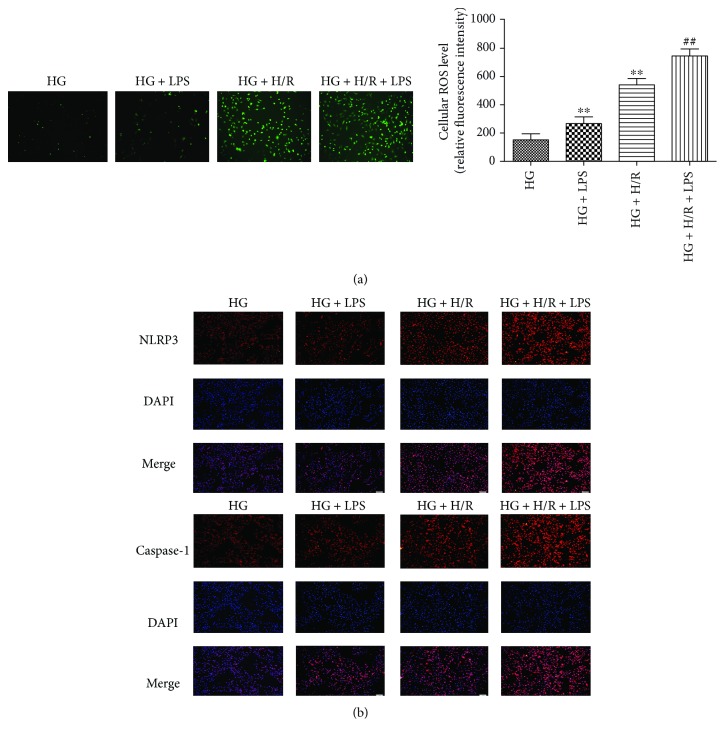
Effects of LPS on ROS production and expressions of NLRP3 and caspase-1 in H9C2 cells subjected to high glucose (HG) and hypoxia/reoxygenation (H/R) insult. The ROS production was detected by DCFH-DA assay (a). The expression of NLRP3 and caspase-1 (b) in H9C2 cells were detected by Immunofluorescence staining. ^∗∗^*P* < 0.01 versus HG; ^##^*P* < 0.01 versus HG+H/R.

To investigate the roles of ROS and the NLRP3 inflammasome in LPS-aggravated H9C2 cell injury under HG and H/R stimulation, we next measured the production of cellular ROS, NLRP3, and caspase-1 protein expression in H9C2 cells. The results showed that the production of cellular ROS was increased under HG+H/R conditions compared with in HG group and further increased by 1 *μ*g/ml LPS treatment (Figure 3(a)). Subsequently, the protein expression of NLRP3 (Figure 3(b)) and caspase-1 (Figure 3(b)) was determined by immunofluorescence staining. As shown in Figures 3(b), H/R stimulation could significantly increase the expression of NLRP3 and caspase-1 in HG condition and further increased by LPS treatment in HG+H/R-stimulated H9C2 cells. These results indicated that LPS increased ROS production then induced the activation of NLRP3 inflammasome in the HG+H/R-induced cell injury in H9C2 cells.

### 3.4. LPS Aggravated HG+H/R-Induced H9C2 Cell Injury by NLRP3 Inflammasome-Mediated Pyroptosis

**Figure 4:**
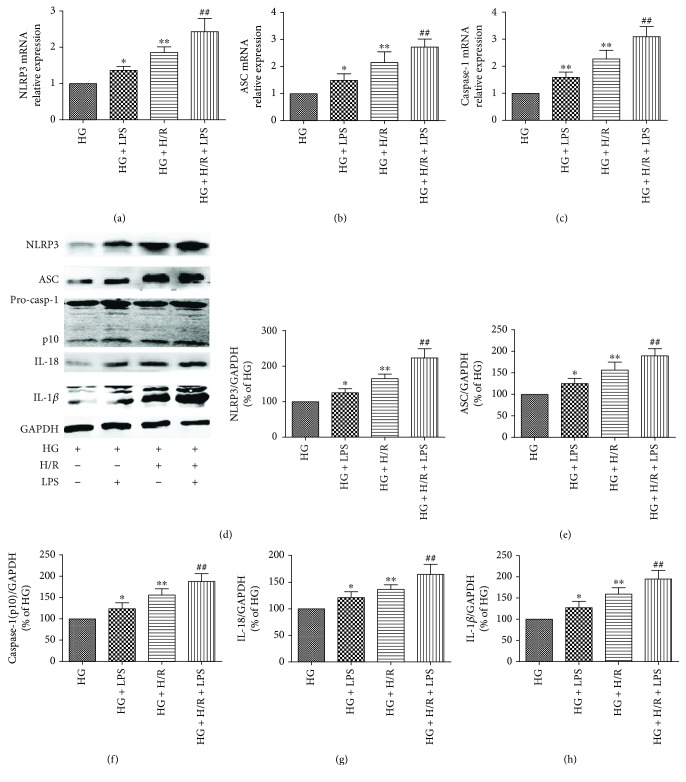
Effects of LPS on NLRP3 inflammasome activation and pyroptosis-related protein expression in HG- and H/R-stimulated H9C2 cells. The mRNA expression of NLRP3 (a), ASC (b), and caspase-1 (c) was detected by real-time PCR. Western blotting for NLRP3 (d), ASC (e), caspase-1 (p10) (f), IL-18 (g), and IL-1*β* (h) in H9C2 cells. Data are expressed as mean ± SD (*n* = 6). ^∗^*P* < 0.05 and ^∗∗^*P* < 0.01 versus HG; ^##^*P* < 0.01 versus HG+H/R.

To investigate the role of NLRP3 inflammasome-mediated pyroptosis in LPS-aggravated H9C2 cell injury under HG and H/R stimulation, we next measured mRNA levels of NLRP3, ASC, and capase-1 and protein expression of NLRP3 inflammasome and pyroptosis-related proteins in H9C2 cells. As the results show, LPS could activate NLRP3 inflammasome characterized by increasing the mRNA levels of NLRP3, ASC, and capase-1 compared with that of HG groups (Figures 4(a)–4(c)) and further increased by LPS treatment in HG+H/R-stimulated H9C2 cells compared with HG+H/R group. As shown in Figures 4(d) and 4(e), H/R stimulation could significantly increase the protein expression of NLRP3 and ASC in HG condition and further increased by LPS treatment in H9C2 cells. What is more, LPS-stimulated H9C2 cells with HG+H/R conditions has significantly increased cleaved caspase-1 (p10), IL-18, and IL-1*β* protein levels compared with HG+H/R group (Figures 4(f)–4(h)). All these results suggested that NLRP3 inflammasome-induced pyroptosis and inflammatory pathway were activated in cultured H9C2 cells under HG and H/R conditions and further increased by LPS stimulation.

### 3.5. Inhibition of NLRP3 Inflammasome and ROS Production Attenuated Cell Injury and Pyroptotic Cell Death in LPS-Treated H9C2 Cells under HG and H/R Conditions

**Figure 5:**
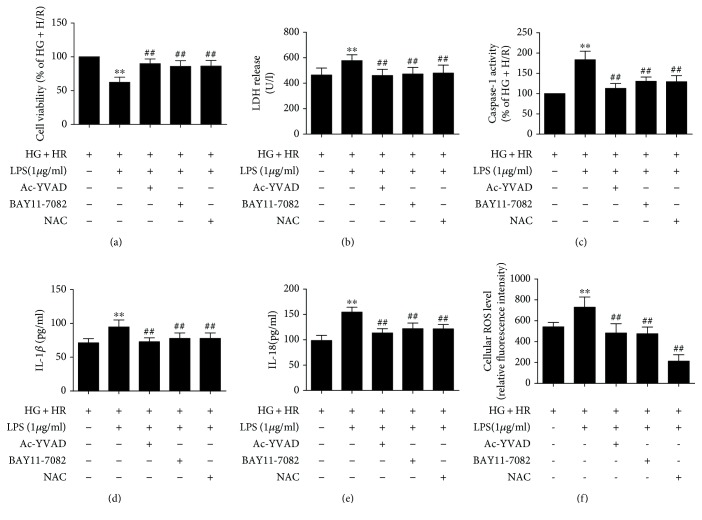
Effects of inflammasome inhibitor and ROS inhibitor on LPS-induced H9C2 cell injury under HG and H/R conditions. Cell viability was detected by CCK-8 assay (a). The LDH release was detected by LDH activity assays (b). Caspase-1 activity was detected by caspase-1 activity assay kit (c). The levels of IL-1*β* (d) and IL-18 (e) in the supernatants were analyzed by ELISA. The ROS production was detected by DCFH-DA assay (f). Data are expressed as mean ± SD (*n* = 6). ^∗∗^*P* < 0.01 versus HG + H/R; ^##^*P* < 0.01 versus HG+H/R+LPS.

In the next experiments, we used caspase-1 inhibitor Ac-YVAD-CMK, inflammasome inhibitor BAY11-7082, and ROS inhibitor NAC to investigate the roles of ROS and NLRP3 inflammasome in LPS-aggravated HG+H/R-induced cell injury in H9C2 cells. As shown in Figures 4(a) and 4(b), Ac-YVAD-CMK and BAY11-7082 significantly reversed the decreased cell viability and increased LDH release by LPS treated in HG+H/R insult H9C2 cells, in accord with attenuated caspase-1 activity (Figure 5(c)). As shown in Figures 5(d) and 5(e), the levels of IL-1*β* and IL-18 in the supernatants were significantly decreased by Ac-YVAD-CMK and BAY11-7082 inhibitors in H9C2 cells. As shown in Figure 5(f), ROS production was significantly decreased by Ac-YVAD-CMK and BAY11-7082 inhibitors in H9C2 cells. In addition, inhibition of ROS production by NAC also reversed LPS-aggravated H9C2 cell HG+H/R injury by increasing cell viability and decreasing LDH release and caspase-1 activity, levels of IL-1*β* and IL-18 in the supernatants, implying that the protective effects of BAY11-7082 and NAC against LPS-aggravated HG+H/R-induced injury was mediated by the production of ROS and NLRP3 inflammasome activation to downregulate pyroptosis.

### 3.6. BAY11-7082 And NAC Protect against LPS-Aggravated HG+H/R Injury by Inhibiting NLRP3 Inflammasome-Mediated Pyroptosis through Downregulating the Protein Expression of NF-*κ*B p65, NLRP3, ASC, Caspase-1, IL-1*β*, and IL-18

**Figure 6:**
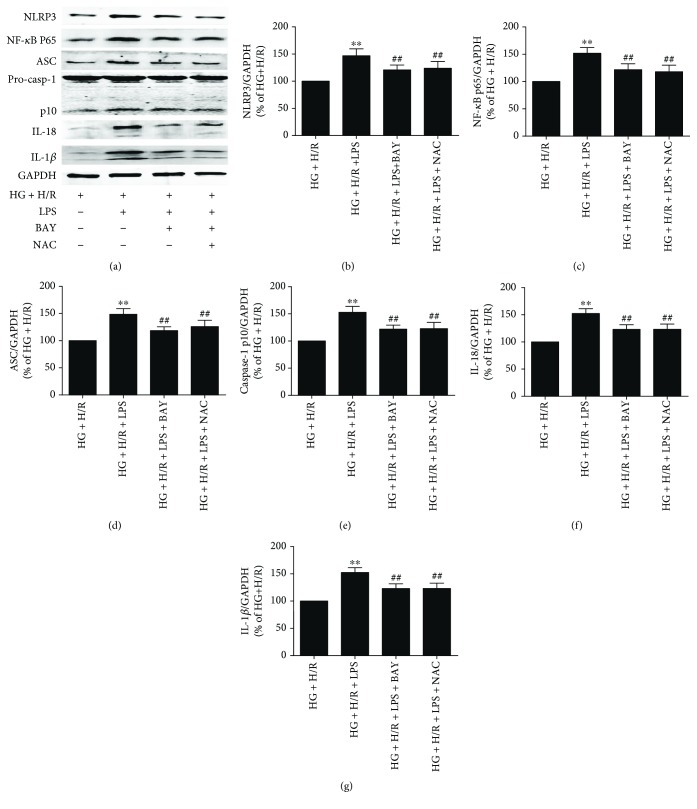
Protein expressions of NLRP3 inflammasome and pyroptotic-related proteins when inflammasome inhibitor and ROS inhibitor in H9C2 cells after LPS stimulated under HG and H/R conditions. Western blotting for NLRP3 (b), NF-*κ*B p65 (c), ASC (d), caspase-1 (p10) (e), IL-18 (f), and IL-1*β* (g) in H9C2 cells. Data are expressed as mean ± SD (*n* = 6). ^∗∗^*P* < 0.01 versus HG+H/R; ^##^*P* < 0.01 versus HG+H/R+LPS.

It is well known that NLRP3, ASC, and caspase-1 multiprotein complex NLRP3 inflammasome is the main pathway of pyroptosis and inflammation in cell injury. We further explored the specific molecular mechanisms that BAY and NAC inhibit LPS-induced HG+H/R injury in H9C2 cells. As shown in Figures 6(a)–6(d), BAY11-7082 and NAC inhibited the NLRP3 inflammasome activation by downregulating the protein levels of NF-*κ*B p65, NLRP3, and ASC in LPS and HG+H/R-treated H9C2 cells. Cleaved caspase-1 (p10) (Figure 6(e)) and mature IL-18 (Figure 6(f)) and IL-1*β* (Figure 6(g)) protein expression were also significantly downregulated by the two inhibitors. These results showed that BAY11-7082 and NAC can alleviate LPS-induced cell injury by decreasing NLRP3 inflammasome activation, reducing activated caspase-1-induced pyroptosis and inflammatory response in HG and H/R conditions.

## 4. Discussion

Patients with diabetes mellitus are at higher risk of cardiovascular events compared with nondiabetic individuals, and diabetic patients are more vulnerable to ischemic heart diseases [1, 2]. Studies have indicated that LPS has been reported to aggravate heart I/R injury and H9C2 cells injury [29, 30]; however, the impact of LPS in HG- and H/R-stimulated cardiomyocytes is unclear. The main finding of our study was that LPS at a 1 *μ*g/ml concentration aggravated HG+H/R-induced injury in H9C2 cells by activating NLRP3 inflammasome activation and pyroptosis through ROS/NLRP3-mediated caspase-1 pathway. Treatment with BAY11-7082 and NAC could protect H9C2 cells from LPS-aggravated HG+H/R injury by inhibiting NLRP3 inflammasome and pyroptosis which was possibly associated with decreased ROS production. To our knowledge, this is the first study to investigate the mechanism about LPS aggravating the HG- and H/R-induced injury in H9C2 cells which was associated with ROS-dependent NLRP3 inflammasome-activated caspase-1 to induce pyroptosis.

Increased oxygen consumption during sepsis may result in a comprehensive acute coronary syndrome, especially when superimposed with preexisting coronary artery disease. Irreversible myocardial infarction occurs when the myocardium lacks oxygen and nutrients during prolonged ischemia [10]. LPS is an antigenic component of the cell wall in Gram-negative bacteria and an activator of TLR4, promoting the upregulation of various inflammatory cytokines that play a key role in the induction and regulation of inflammatory responses [31]. The combination of LPS and its receptor activates the overproduction of proinflammatory mediators, resulting in myocardial damage, heart failure, and even death [9]. However, the role of LPS in myocardial I/R injury is still controversial. Studies have reported that pretreatment of mice with low-dose LPS significantly reduced myocardial infarction following I/R [32, 33]. On the other hand, other studies have suggested that LPS may be involved in regulating glucose homeostasis by stimulating glucagon and insulin release [34, 35] and have reported that LPS inhibited cell viability, increases ROS levels, and promotes apoptosis to aggravate oxidative damage in H9c2 cells [29, 36, 37]. In Chen et al.'s study, the dose of 15 mg/kg LPS could significantly induce myocardial injury in vivo, and LPS administration at 4 *μ*g/ml concentration could pronouncedly increase the level of ROS in H9C2 [29]. Therefore, different dose of LPS could play different role in myocardial ischemia-reperfusion and myocardial injury. In our study, we used 0.1 *μ*g/ml or 1 *μ*g/ml concentrations of LPS to stimulate H9C2 cardiomyocytes under HG and H/R conditions. As the results show that higher concentration of 1 *μ*g/ml LPS could aggravate HG- and H/R-induced injury in H9C2 cells by decreasing cell viability and increasing LDH level, ROS production, and pyroptotic cell death and accompanied with increased expression of NLRP3, ASC, activated caspase-1 (p10), IL-1*β*, and IL-18. This suggested that higher concentration of LPS can aggravate HG- and H/R-induced injury in H9c2 cells by inducing pyroptosis.

The NLRP3 inflammasome, as the mediator of inflammatory response, has been activated by both PAMPs and DAMPs, such as LPS, ATP, glucose, ROS, and crystallisation [12, 38, 39]. The activation of NLRP3 inflammasome could cleave the pro-caspase-1 to activated caspase-1, which then led to the maturation of inflammatory cytokine IL-1*β* and IL-18. The molecular mechanisms of NLRP3 inflammasome activation are complex and currently considered to be broadly divided into three major pathways: the first pathway is that extracellular ATP stimulates P2X7-dependent ion channel opening to promote the formation of K^+^ efflux and connexin membrane channels then to directly promote aggregation and activation of NLRP3 inflammasome. The second route is that extracellular crystals or special particles are endocytosed into the cells and cause lysosomal rupture to promote the aggregation and activation of NLRP3 inflammasome. The third pathway is that PAMPs and DAMPs promote the increase of intracellular ROS production and promote the aggregation and activation of NLRP3 inflammasome through ROS-dependent signaling pathways [12]. In the present study, we found that LPS increased the ROS production and NLRP3 inflammasome activation and further increased the expression of activated caspase-1 (p10), IL-1*β*, and IL-18 in HG condition of H9C2 cells. In addition, under HG+H/R condition, the LPS increased the activation of NLRP3 inflammasome and the expression of activated caspase-1 (p10), IL-1*β* and IL-18 in H9C2 cells to aggravated HG- and H/R-induced injury. What is more, when inflammasome inhibitor BAY11-7082 was used, the LPS-aggravated HG+H/R-induced injury was relieved by downregulating NLRP3 inflammasome. These results provided evidence that LPS aggravated HG-and H/R-induced injury in H9C2 cells by activating NLRP3 inflammasome which is an important signaling pathway to induce pyroptosis and inflammatory reaction.

Pyroptosis is a caspase-1-dependent cell death, and is considered to play a crucial role in the dysregulation of inflammatory/immune responses in sepsis [40–42]. Pyroptosis is induced by the two distinct stimuli, microbial PAMPs and endogenous DAMPs. In response to DAMPs, NLRP inflammasome is formed, where caspase-1 is activated, and finally, active caspase-1 processes/releases IL-1*β* to induce cell death [41]. Pyroptosis is advantageous for host defense against intracellular pathogens, which is convenient to kill damaged cell as a result. On the other hand, pyroptosis is important in the inflammatory process since it can make activated macrophages rapidly release large amounts of cytokines into the extracellular space, and pyroptosis-induced depletion of immune cells is associated with magnified inflammatory cascade amplification [43, 44]. Studies have indicated that hyperglycaemia increased the production of ROS to trigger the caspase-1-dependent pyroptosis which mediates important pathological changes in diabetic cardiomyopathy [45, 46]. Our previous study have also found that caspase-1-dependent pyroptosis induced by NLRP3 inflammasome activation contributed to diabetic myocardium and aggravated MI/R injury in diabetes [27]. In this study, we utilized LPS as a PAMP to stimulate H9C2 cardiomyocytes in HG and H/R conditions. The results indicated that LPS increased inflammatory and pyroptosis by upregulating the activation of NLRP3 inflammasome and the expression of cleaved caspase-1p10, IL-1*β*, and IL-18. Furthermore, when we used caspase-1 inhibitor Ac-YVAD-CMK and inflammasome inhibitor BAY11-7082 to inhibit caspase-1 and NLRP3 inflammasome, the LPS-induced cell injury in H9C2 cells exposed to HG and H/R conditions was significantly decreased. Taken together, our results demonstrated that LPS-induced activation of NLRP3-mediated caspase-1-dependent pyroptosis may play an important role in aggravated H9C2 cells HG and H/R injury.

ROS acts as second messenger to drive inflammasome activation and has been identified as an important mechanism for NLRP3 inflammasome activation [47] and is believed to be a common NLR/caspase-1 complex activator, which mediates pyroptosis [48]. Under normal conditions, the antioxidant enzymes can eliminate ROS during cell metabolism to maintain a balance of ROS generation and elimination [49]. When ROS accumulates ceaselessly and endogenic antioxygen defence system cannot eliminate it in time, it induces oxidative stress, cellular disorders including upregulated lipid peroxidation, and cell apoptosis [50]. New researches suggest that LPS induces cardiomyocyte injury mostly through excessive ROS generation [51, 52]. ROS can activate the expression of NF-*κ*B which is a crucial transcription factor in inflammation, stress response, and cell growth and survival [53]. The hyperglycaemia, hyperinsulinaemia and insulin resistance of diabetes enhanced oxidative stress and led to excessive cytokine generation in the diabetic myocardium. Moreover, when MI/R occurs, ion channel opening (such as K^+^ efflux) increases the production of ROS to induce inflammatory cascades. In the present study, we found that the NLRP3 inflammasome activation significantly increased in a ROS-dependent manner to induce pyroptotic cell death in HG and H/R conditions with LPS stimulation. We also found that NAC inhibited ROS production to decrease the activation of NLRP3 inflammasome then to alleviate LPS-induced HG and H/R injury in H9C2 cells. These results indicated that ROS is an activator for NLRP3 inflammasome and caspase-1-dependent pyroptosis in LPS-aggravated H9C2 cell injury within HG and H/R conditions. Moreover, inhibition of ROS is an effective treatment to attenuate LPS-aggravated H9C2 cells HG and H/R injury.

In conclusion, our findings suggested that exposure to high concentration (1 *μ*g/ml) of LPS could increase the sensitivity of H9C2 cells to HG and H/R. LPS aggravated H9C2 cells HG- and H/R-induced injury by targeting ROS/NLRP3 inflammasome pathway and the downstream activated caspase-1 and IL-1*β* pathway to mediate pyroptosis and inflammatory responses. Our results also confirmed that inflammasome inhibitor or ROS scavenger NAC can attenuate LPS-increased HG+H/R-induced injury in H9C2 cells by inhibiting the ROS/NLRP3 inflammasome activation to reduce pyroptosis, which may be a new therapeutic target to mitigate myocardial ischemia/reperfusion injury during sepsis. However, the specific mechanisms of LPS-induced ROS/NLRP3 inflammasome activation and pyroptosis to increase the sensitivity to HG and H/R stimulation remain to be further addressed.

## Data Availability

The data used to support the findings of this study are included within the article.
